# On the evolution of the company we keep: Implications for infectious disease modeling

**DOI:** 10.1371/journal.pmed.1005075

**Published:** 2026-05-13

**Authors:** Joël Mossong

**Affiliations:** Health Directorate, Ministry of Health and Social Security, Luxembourg, Luxembourg

## Abstract

In this Perspective, Joël Mossong discusses the importance of understanding social contacts and how they have evolved for infectious disease modeling, and the need to factor in additional considerations such as ethic and socioeconomic backgrounds.

## Teaser

Whom we meet shapes how infections spread. Where earlier focus of mathematical epidemiology was on incorporating age, more recent work has begun to reveal the importance of socioeconomic aspects for understanding and managing future epidemics.

Infectious disease transmission is, at its core, a social phenomenon. Respiratory pathogens travel along the lines of everyday human interaction—between children in classrooms, colleagues in workplaces, and families at home—yet for many years in mathematical epidemiology, the structure of those interactions was largely assumed rather than measured.

Pioneering work by Anderson and May, synthesized in their 1991 book, formalized age-structured transmission using the “Who Acquires Infection From Whom” (WAIFW) matrices [1]. These matrices elegantly captured assortative mixing and heterogeneity, but they were usually parameterized through indirect inference or pragmatic simplification. In the 1990s, John Edmunds and colleagues began exploring whether contact patterns could be measured empirically. In the first pilot study [2], contact was defined as a “two-way conversation (at a distance not requiring raised voices) in which at least two words were spoken by each party and where no physical barrier separated them”.

A decisive advance came with the EU-funded POLYMOD study, published in *PLOS Medicine* in 2008 [3]. POLYMOD provided, for the first time, large-scale, standardized diary-based contact data across eight European countries. One of the main challenges was ensuring strict adherence to a common methodology across all participating countries. Nonetheless, the study helped establish empirically derived contact matrices as standard inputs for mathematical transmission models, as reflected in its widespread adoption within the modeling community.

Then came the COVID-19 pandemic. It transformed contact measurement from a specialized research activity into a policy necessity. Here, the Imperial College modeling report by Ferguson and colleagues (published March 2020), which helped precipitate the first UK lockdown [4], brought transmission modeling into the center of political decision-making. It used age-structured contact matrices (largely derived from the POLYMOD study) to simulate viral transmission under different intervention scenarios. In addition, the CoMix study was conducted to provide repeated estimates of social mixing during periods of restriction and recovery [5,6]. This is reflected in UK advisory analyses from the Scientific Advisory Group for Emergencies (SAGE) in April 2021, which emphasized that the impact of nonpharmaceutical interventions depends critically on patterns of social contact, behavioral adherence, and the combined effects of intervention packages rather than individual measures alone. [7]

Yet criticism soon followed that early models only considered age heterogeneity but lacked an explicit socioeconomic dimension. The UK COVID-19 inquiry repeatedly highlighted the disproportionate burden borne by vulnerable and disadvantaged populations [8]. In the first 5 months of the pandemic, Black African men were twice as likely to die from COVID-19 than White men, even after adjusting for age, geography, and socioeconomic status [9].

The pandemic thus served as a reminder that social stratification—“class”, in the broadest sense—remains a defining feature of our societies, and therefore of the epidemics that move through them.

In a recent *PLOS Medicine* article, the Reconnect study by Goodfellow and colleagues revisits this line of work in a post-pandemic context [10]. Their survey shows that, after the pandemic, people reported an average of 9.1 contacts per day—a 20% decrease from 2006, but a 40% increase relative to late 2022. Perhaps the most important advance is that social mixing was estimated by ethnicity and socioeconomic status, revealing assortativity within households and workplaces and quantifying differential infection risk. Their modeling work suggests that Black individuals would experience a 2.3 higher infection risk than White people in the event of novel respiratory virus [10].

Integrating socioeconomic contact patterns into models could improve their policy relevance. In particular, such models could help identify which population groups—defined jointly by age and socioeconomic characteristics—would benefit most from targeted medical and nonmedical interventions, such as prioritization for testing, vaccination, or other tailored public health measures.

This study also illustrates how contact patterns have evolved over the past two decades. The increasing use of smartphones and digital platforms had already shifted many interactions into virtual space before 2020. The pandemic accelerated these trends: remote working, online shopping, and reduced commuting have altered who we meet face to face, and therefore with whom we potentially exchange respiratory pathogens. The company we keep has changed, and so too has the epidemiological landscape.

What is also clear is that future modeling efforts need to incorporate a socioeconomic dimension alongside age structure. Until recently, such data were limited, but reports such as the Reconnect study now provide detailed empirical evidence on how social contacts vary across socioeconomic groups. This opens new opportunities to better parameterize mathematical transmission models. These patterns are unlikely to be uniform across countries and settings: demographic composition, household structures, and social inequalities differ substantially, meaning that findings from the UK cannot be easily generalized elsewhere.

There is therefore much still to learn. Social contacts are not static; they evolve with technology, economics, and policy. Understanding how they change over time and what those changes mean for disease transmission, population immunity, and disease prevention will remain a key area of research in infectious disease epidemiology.
